# Predicting OCT images of short-term response to anti-VEGF treatment for retinal vein occlusion using generative adversarial network

**DOI:** 10.3389/fbioe.2022.914964

**Published:** 2022-10-12

**Authors:** Fabao Xu, Xuechen Yu, Yang Gao, Xiaolin Ning, Ziyuan Huang, Min Wei, Weibin Zhai, Rui Zhang, Shaopeng Wang, Jianqiao Li

**Affiliations:** ^1^ Department of Ophthalmology, Qilu Hospital, Cheeloo College of Medicine, Shandong University, Jinan, China; ^2^ School of Physics, Beihang University, Beijing, China; ^3^ Hangzhou Innovation Institute, Beihang University, Hangzhou, China; ^4^ Research Institute of Frontier Science, Beihang University, Beijing, China; ^5^ Zibo Central Hospital, Binzhou Medical University, Zibo, Shandong, China

**Keywords:** deep learning, retinal vein occlusion, generative adversarial networks, optical coherence tomography, artificial intelligence

## Abstract

To generate and evaluate post-therapeutic optical coherence tomography (OCT) images based on pre-therapeutic images with generative adversarial network (GAN) to predict the short-term response of patients with retinal vein occlusion (RVO) to anti-vascular endothelial growth factor (anti-VEGF) therapy. Real-world imaging data were retrospectively collected from 1 May 2017, to 1 June 2021. A total of 515 pairs of pre-and post-therapeutic OCT images of patients with RVO were included in the training set, while 68 pre-and post-therapeutic OCT images were included in the validation set. A pix2pixHD method was adopted to predict post-therapeutic OCT images in RVO patients after anti-VEGF therapy. The quality and similarity of synthetic OCT images were evaluated by screening and evaluation experiments. We quantitatively and qualitatively assessed the prognostic accuracy of the synthetic post-therapeutic OCT images. The post-therapeutic OCT images generated by the pix2pixHD algorithm were comparable to the actual images in edema resorption response. Retinal specialists found most synthetic images (62/68) difficult to differentiate from the real ones. The mean absolute error (MAE) of the central macular thickness (CMT) between the synthetic and real OCT images was 26.33 ± 15.81 μm. There was no statistical difference in CMT between the synthetic and the real images. In this retrospective study, the application of the pix2pixHD algorithm objectively predicted the short-term response of each patient to anti-VEGF therapy based on OCT images with high accuracy, suggestive of its clinical value, especially for screening patients with relatively poor prognosis and potentially guiding clinical treatment. Importantly, our artificial intelligence-based prediction approach’s non-invasiveness, repeatability, and cost-effectiveness can improve compliance and follow-up management of this patient population.

## Introduction

Retinal vein occlusion (RVO) represents the second most common cause of vision loss worldwide due to retinal vasculopathy, predominantly affecting the middle-aged and elderly (Wong and Scott, 2010; Ip and Hendrick, 2018; Fang et al., 2021; Blair and Czyz, 2022). Macular edema is the most common cause of RVO-related vision loss, characterized by fluid accumulation within the central retina and macular thickening caused by blood-retinal barrier dysfunction (Khayat et al., 2018; Korobelnik et al., 2021). Except for a minority of patients with non-ischemic RVO, most cases present with RVO-related macular edema, leading to irreversible visual loss, poor quality of life and substantial socioeconomic burden if proper and timely treatment is not provided (Loukianou et al., 2016; Lee et al., 2021a; Korobelnik et al., 2021).

The primary goal of treating RVO-related macular edema is to reduce the fovea’s central macular thickness (CMT) and maintain the central visual acuity, which involves reducing the accumulation of the inner retinal fluid (Sangroongruangsri et al., 2018; Lee et al., 2021a). In recent years, the treatment of macular edema has become a research hotspot in the field of ocular fundus diseases, and various new treatment methods have been implemented (Chen et al., 2022). The first-line treatment for RVO-related macular edema consists of intravitreal injections of anti-Vascular Endothelial Growth Factor (anti-VEGF) (Fogli et al., 2018; Korobelnik et al., 2021). In most cases, anti-VEGF therapy can reduce fluid accumulation and improve visual acuity (Korobelnik et al., 2021; Wallsh and Gallemore, 2021). However, not all patients respond well to anti-VEGF therapy (Korobelnik et al., 2021). Given the differences in drug regimen and patient characteristics across treatment groups, it is difficult to predict individual treatment responses before patients receive anti-VEGF therapy, even for experienced retinal specialists (Gallardo et al., 2021; Park et al., 2021). Optical coherence tomography (OCT) is widely acknowledged as a high-resolution imaging modality for quantifying retinal thickening and fluid accumulation in patients with RVO and assessing the severity of macular edema. Accordingly, OCT is the primary tool for the examination and follow-up of RVO-related macular edema cases (Kashani et al., 2017; Song et al., 2021).

Among non-surgery-related blindness-causing retinopathies, RVO ranks second in incidence after diabetic retinopathy (DR) and second to diabetic macular edema as the cause of inner retinal fluid accumulation (Xu et al., 2021a; Hayreh, 2021). The broad application of fundus fluorescein angiography (FFA) and OCT in RVO-related macular edema has significantly improved our understanding of its pathogenesis and provides an opportunity to collect large-scale real-world imaging data (Sandmeyer et al., 2022). Meanwhile, the application of artificial intelligence (AI) in the medical field has become increasingly popular in recent years (Caixinha and Nunes, 2017). The past decade has witnessed several inroads achieved with AI being harnessed for learning and mining fundus image data, assisting doctors in screening, diagnosing, and treating various retinopathies (Xu et al., 2021b; Ting et al., 2021). Generative adversarial network (GAN), first proposed by Ian Goodfellow in 2014, is an AI-based “image-to-image” algorithm that can synthesize new images based on existing ones (Goodfellow et al., 2016; Xu et al., 2021a). The core principle of GANs is to generate fake data that closely resembles real data (Kazeminia et al., 2020). In recent years, the GAN-based algorithm has yielded satisfactory performance when used to predict the effect of anti-VEGF therapy or laser photocoagulation for neovascular age-related macular degeneration (nAMD) and central serous chorioretinopathy (CSC) (Liu et al., 2020a; Xu et al., 2021a). Given the high incidence of RVO-related macular edema, the difficulty in predicting the therapeutic effect, the burden of anti-VEGF therapy, and the frequency of follow-ups, it is essential to develop a novel approach for individualized prediction of the therapeutic effect (Wecker et al., 2017). Accordingly, this study aimed to generate and evaluate individualized post-therapeutic OCT images that could predict the short-term response of anti-VEGF therapy for RVO-related macular edema based on pre-therapeutic images using a GAN-based algorithm.

## Materials and methods

### Clinical data and imaging examinations

To predict the short-term response to anti-VEGF therapy and generate individualized post-therapeutic OCT images based on pre-therapeutic images using a GAN-based model, we retrospectively reviewed the records of patients with RVO-related macular edema who underwent intravitreal injection of anti-VEGF drugs at the Department of Ophthalmology, Qilu Hospital, Shandong University from 1 May 2017, to 1 June 2021. The inclusion criteria consisted: 1) patients aged ≥ 18 years; 2) patients with a pre-operative diagnosis of RVO based on fundus photography and fundus fluorescein angiography (FFA) and further examination based on OCT; and 3) patients treated with an injection of anti-VEGF including conbercept or ranibizumab, at any phase in the treatment protocol of three consecutive monthly injections and pro re nata (PRN) injections. The exclusion criteria were: 1) presence of any other retinal and/or choroidal diseases, including diabetic retinopathy (DR), age-related macular degeneration (AMD), and polypoidal choroidal vasculopathy (PCV), which may affect the study; 2) history of surgery, or intraocular injections of medications other than anti-VEGF agents; 3) history of other ocular disorders, including glaucoma, pathological myopia; and 4) low image quality caused by media opacities, or an abnormal signal strength index on the OCT images. The follow-up visit was scheduled at 1 month after the intraocular injection. However, it was difficult for the ophthalmologist to perform the follow-up on fixed dates due to the different work schedules of RVO patients. Accordingly, we determined a time range for the follow-up to ensure data accuracy: 1 month ± 7 days after anti-VEGF therapy. All OCT images enrolled were stripped of personally identifiable information. The need for written informed consent was waived by our ethics committee due to the retrospective nature of the study, and all data used were fully anonymized. Moreover, this study was conducted based on the ethical principles of the Declaration of Helsinki and approved by the institutional review board of Qilu Hospital [Ethical Code: 2021 (068)].

### Data collection

Pre-and post-therapeutic B-scan swept-source OCT (Zeiss, Germany) images were obtained in a 21-line 9 mm macula pattern using the follow-up mode, which enabled the paired pre-therapeutic and post-therapeutic images to be scanned at the same location. OCT images were section-matched based on the retinal microstructure, including the position of the fovea and retinal vessels. OCT images of different layers obtained from the same patient with macular edema were included in the study to ensure optimal use of the image resources of patients with RVO (Supplementary Figure S1). To prepare OCT image pairs for GAN model training, every pre-therapeutic OCT image was paired with the corresponding post-therapeutic OCT image of the same patient. For convenience of model training, image pre-processing was conducted to resize the images. OCT images with an original resolution of 1,264 × 596 pixels were cropped to obtain images of 760 × 490 pixels and further resized into 512 × 512 pixels, the unified format for input during model training.

To ensure that the data of the training set and validation set were not duplicated, we divided the training set and validation set by date. The total number of B-scan OCT image pairs was 583; 515 pairs collected from May 2017 to October 2020 were attributed to the training set for model training, and the remaining 68 OCT image pairs from October 2020 to June 2021 were used as the validation set for model evaluation.

### Image synthesis

**FIGURE 1:**
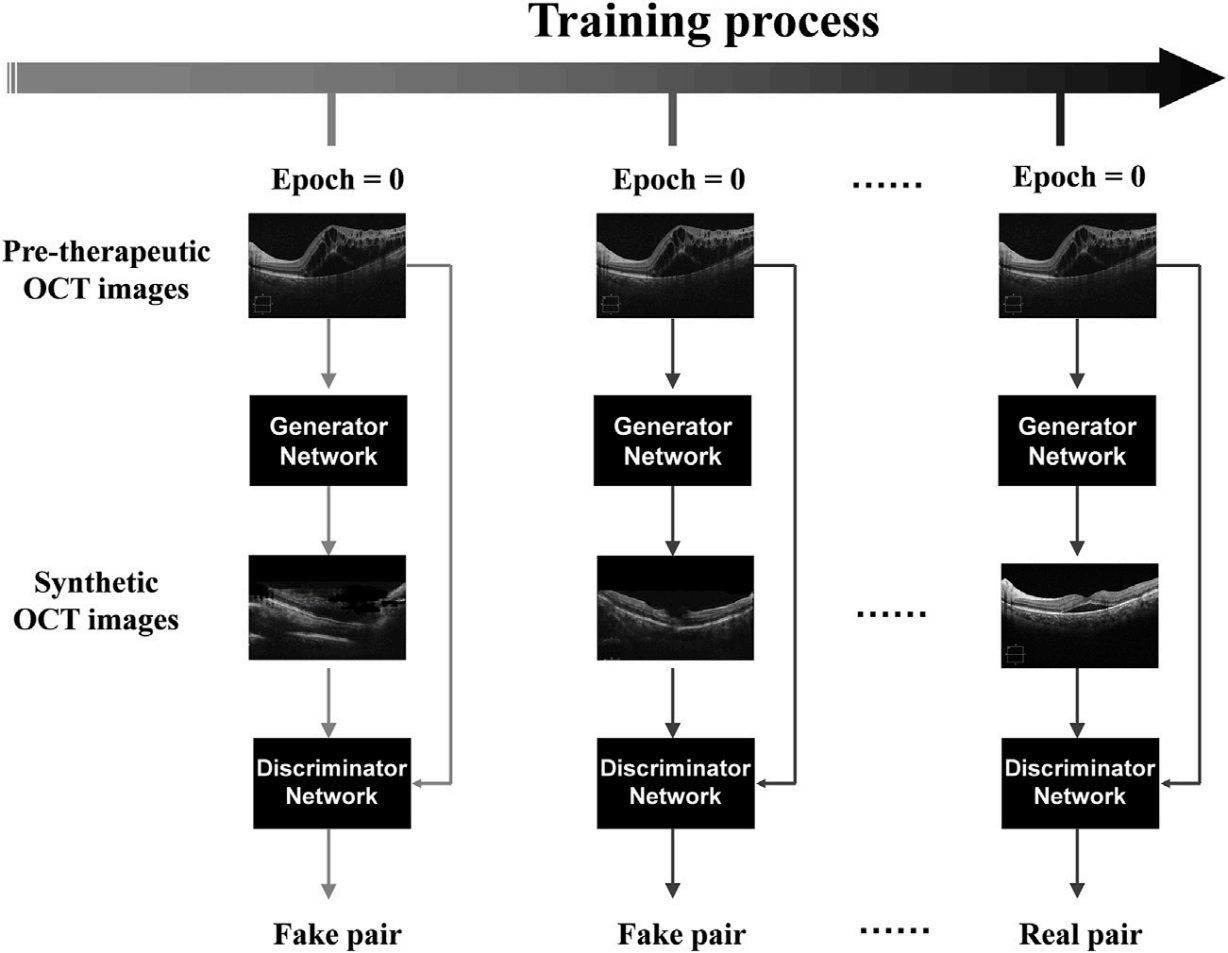
The training process of pix2pixHD. Illustration of the pix2pixHD-based solution used in this study for predicting post-therapeutic OCT images from pre-therapeutic OCT images. OCT, optical coherence tomography.

**FIGURE 2:**
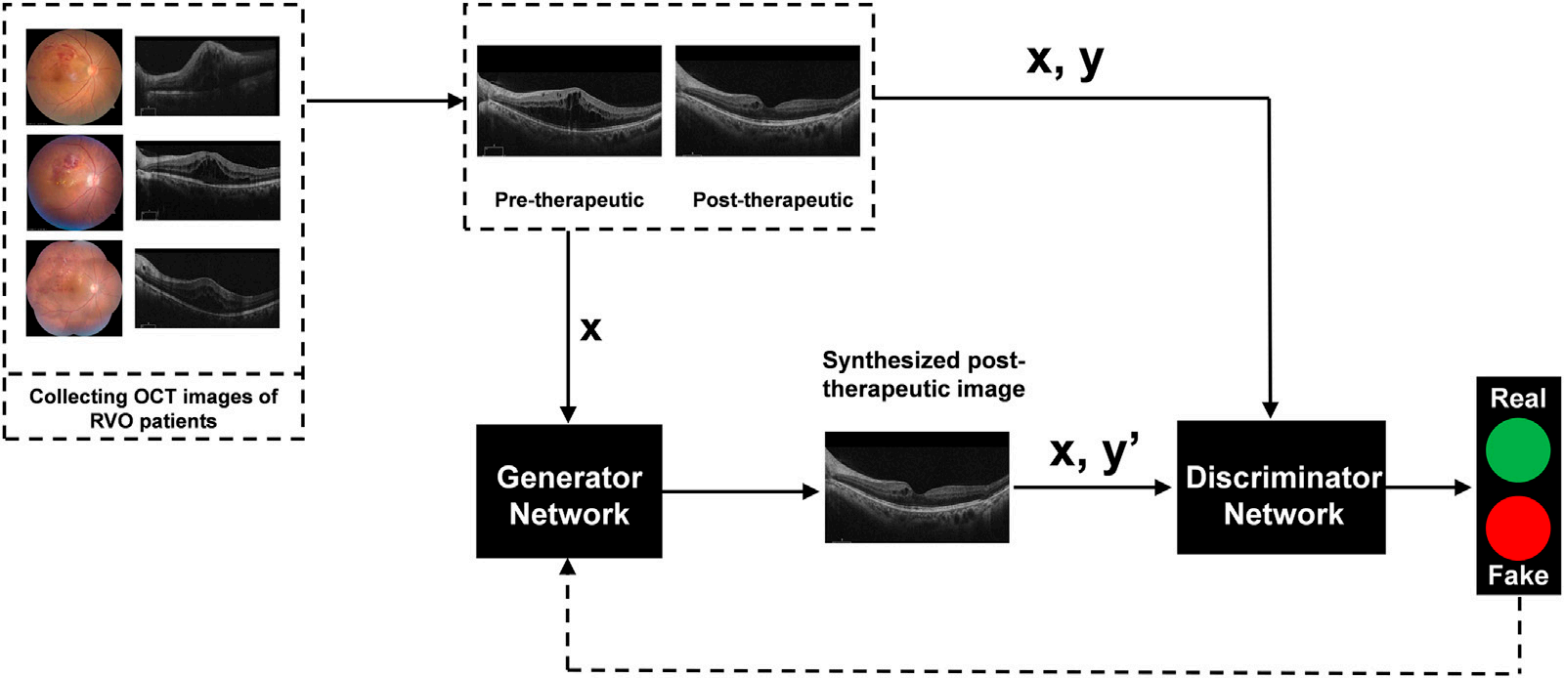
Illustration of generating post-therapeutic OCT from pre-therapeutic OCT by the GAN-based algorithm. OCT, optical coherence tomography; GAN, generative adversarial networks; RVO, retinal vein occlusion.

The generative adversarial network (GAN)-based algorithms were successfully applied in the model training process to establish a deep learning model capable of generating post-therapeutic OCT images based on pre-therapeutic ones (Li et al., 2020; Tschuchnig et al., 2020). As one of the most widely used algorithmic paradigms in deep learning, GAN-based algorithms involve an iterative training process to generate almost realistic data. It has been established that the overall framework of GAN mainly includes two deep neural networks as players: a generator network and a discriminator network. While the generator net is designed to learn mapping from pre-therapeutic images to post-therapeutic ones, the discriminator net distinguishes between real post-therapeutic images and generated post-therapeutic ones. These two networks are trained simultaneously in an adversarial learning process: the generator net iteratively updates itself to generate near-realistic images that are difficult to distinguish by the discriminator net, while the discriminator net constantly updates itself to ensure that near-realistic images can be distinguished from the real ones. After this game-playing process is terminated, an equilibrium is achieved, and the final generator net is expected to generate OCT images almost close to real post-therapeutic ones. During the training process in our study, Adam was used as the optimizer and the momentum term of Adam was set to 0.5. The learning rate was set to 0.0002, the number of iterations to 150, and the number of iterations to linearly decay the learning rate to zero to 150. During model training, we implemented pix2pixHD using Python and Pytorch on Ubuntu 16.04 LTS with GeForce GTX 2080 Ti. The training process of pix2pixHD is shown in Figure 1. The overall training process is displayed in Figure 2.

During model training, the parameter settings were set as follows. For the deep neural network optimizer, we used Adam (Adaptive Moment Estimation, a widely accepted improvement of SGD (Stochastic Gradient Descent) for deep neural network optimizing) as the optimizer for parameter updating. Furthermore, we used the decay learning rate setting, in which the number of iterations for the linear decay of the learning rate to zero was set to 150, the forgetting factor stochastic gradients to 0.5, and the second moment of the stochastic gradient to 0.999.

### Evaluation of post-therapeutic optical coherence tomography prediction models

To evaluate the performance of the pix2pixHD model, the quality and similarity of synthetic OCT images were evaluated by a screening experiment independently. The screening experiment evaluated the similarity of synthetic post-therapeutic OCT images of patients with RVO. All synthetic images and corresponding real OCT images were presented to two retinal ophthalmologists (Fabao Xu and Xuechen Yu), who independently answered two questions: 1) Is the synthetic image suitable for clinical interpretation; and 2) Can you identify the synthetic image? Only synthetic images of sufficient quality that were difficult to distinguish from the original ones were further analyzed in the evaluation experiment.

To quantitatively evaluate the model performance, we applied two evaluation indicators. First, we measured the CMT of both synthetic OCT images and the real ones. Then we used the mean absolute error (MAE) as the evaluation metric. The MAE is calculated as the average value of the absolute error of the prediction results, directly reflecting the deviation of the predicted values from the actual values. The formula for the MAE is as follows:

$$MAE=\frac{1}{N}\sum_{i=1}^{N}\left|{\tilde{y}}_{i}-y_{i}\right|$$

**FIGURE 3:**
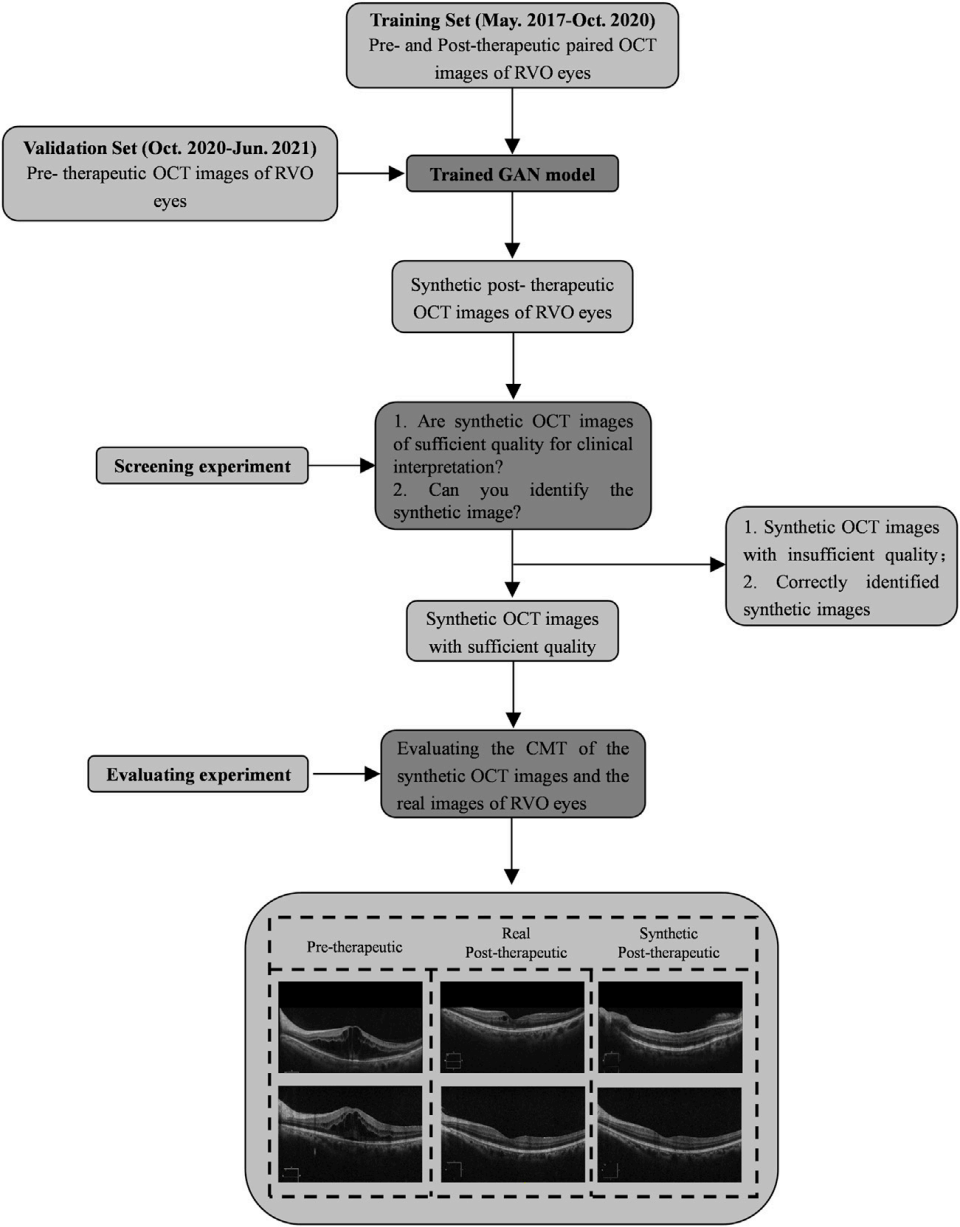
Workflow of evaluating the model performance. OCT, optical coherence tomography; RVO, retinal vein occlusion; GAN, generative adversarial networks; CMT, central macular edema.

The evaluation processes are shown in Figure 3.

## Results

### Demographic data of training and validation data

515 pairs of OCT images from 64 patients were assigned to the training set, and 68 pairs of OCT images from 18 patients to the validation set. 51.56% of eyes in the training group from female patients with a mean age of 53.86 (±13.78) years. In contrast, in the validation set, 55.56% were eyes from female patients with a mean age of 54.16 (±12.54) years. More baseline clinical and demographic data are shown in Table 1. There were no significant differences in age, gender, VA, classification of RVO, Anti-VEGF agent, and injection phase between the training and validation sets. 68 synthetic post-therapeutic images were generated based on pre-therapeutic OCT images of patients with RVO in the validation set.

**TABLE 1:** Patient demographics.

	Training set	Validation set	*p* value
Patients (Female)	64 (33)	18 (10)	N/A
Eyes	64	18	N/A
Ages	53.86 ± 13.78	54.16 ± 12.54	0.917
Paired OCT images	515	68	N/A
VA baseline	0.723 ± 0.215	0.718 ± 0.208	0.821
VA 1-month	0.476 ± 0.325	0.481 ± 0.331	0.863
Classification of RVO			0.989
CRVO	25 (39.06%)	7 (38.89%)	N/A
BRVO	39 (60.94%)	11 (61.11%)	N/A
Anti-VEGF agent (%)			0.771
Ranibizumab	38 (59.38%)	10 (55.56%)	N/A
Conbercept	26 (40.62%)	8 (44.44%)	N/A
Injection phase			0.542
Loading phase	44 (68.75%)	11 (61.11%)	N/A
PRN phase	20 (31.25%)	7 (38.89%)	N/A
Combined with laser photocoagulation			0.645
With laser photocoagulation	5 (7.81%)	2 (11.11%)	N/A
Without laser photocoagulation	59 (92.19%)	16 (88.89%)	N/A

VA, visual acuity, values are presented as the means ± standard deviations at baseline in different groups [in logarithm of minimum angle of resolution (logMAR) units].

### Screening experiment of synthetic images

A total of 68 synthetic OCT images were generated based on pre-therapeutic OCT images by GAN models. The post-therapeutic OCT images predicted by the pix2pixHD model were compared with the ground truth data. During the screening experiment, 3 pairs of synthetic images considered inadequate by ophthalmologist 1 (Fabao Xu) were found adequate by ophthalmologist 2 (Xuechen Yu). Finally, the third specialist (Ying Zhang) was consulted, and one pair was considered inadequate and excluded from the subsequent experiments. In the following experiment designed to distinguish the synthetic OCT images from the real images (ground truth), ophthalmologist 1 accurately identified 5 pairs of synthetic images, while ophthalmologist 2 accurately identified 3 pairs (overlapped with the image identified by ophthalmologist 1) of synthetic OCT images. Most synthetic images (63/68) were challenging to identify by retinal ophthalmologists. Synthetic images that could not be identified were further analyzed in the following evaluation experiment. Examples of inadequate and easily distinguishable synthetic images deleted in the screening experiment are shown in Supplementary Figure S1.

### Evaluation experiment of adequate synthetic images

**FIGURE 4:**
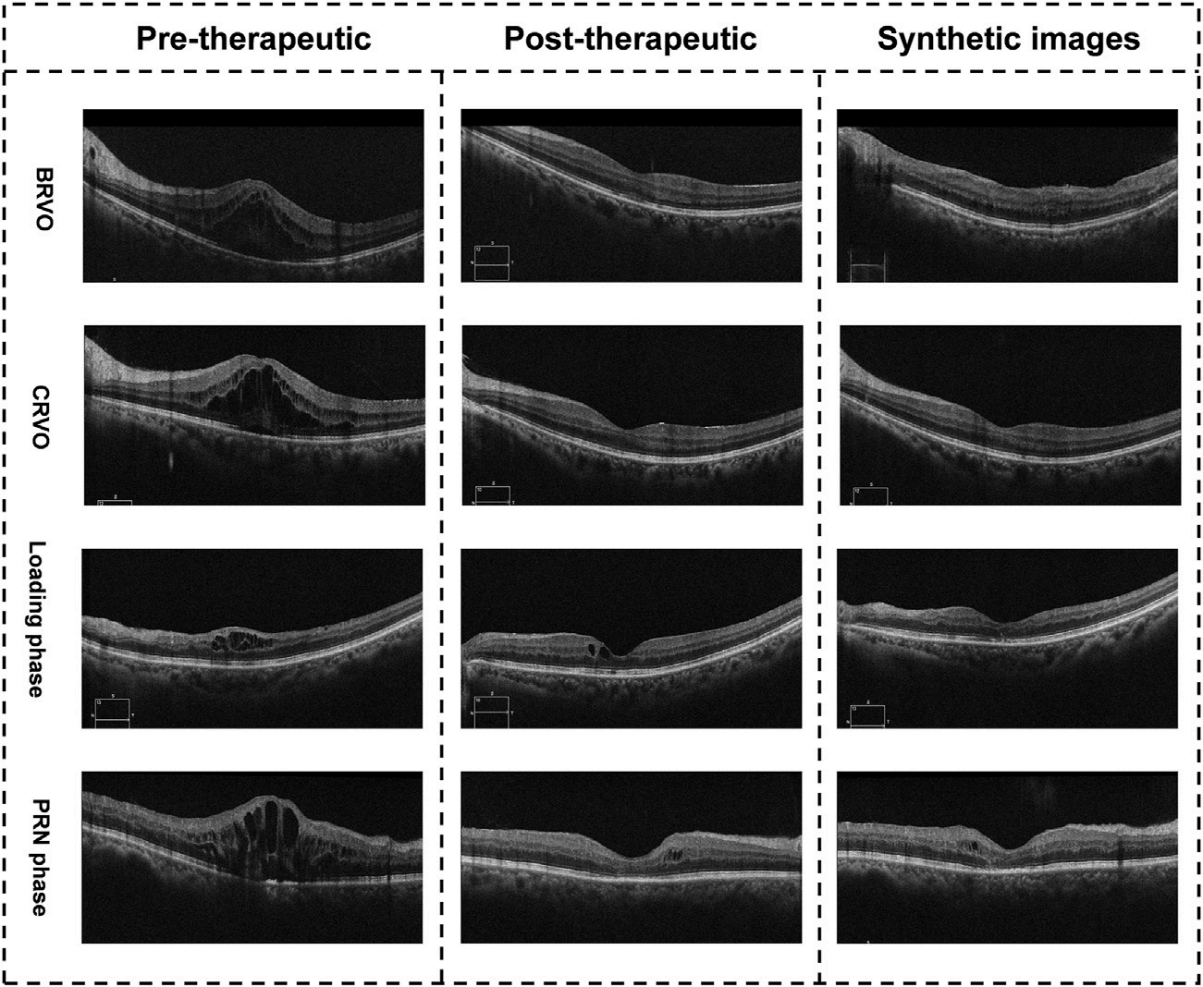
Illustration of the synthetic OCT images with different types of macular edema. The images in the right column are synthetic post-therapeutic images generated by pix2pixHD. The images in the middle column are the real images.

During the evaluation experiment, two retinal specialists (Ying Zhang and Jiawei Wang) measured the CMT of all synthetic post-therapeutic OCT images independently. The mean values of the two measurements were calculated for further analysis. The evaluation experiment included 63 synthetic images from the pix2pixHD algorithm, with an MAE of 26.33 ± 15.81 μm. Illustrations of the synthetic post-therapeutic OCT images with different types of macular edema are shown in Figure 4. In the subgroup analysis of different classifications of RVO-related macular edema, the MAEs of post-therapeutic OCT images from patients with CRVO and BRVO were 28.55 ± 17.32 and 24.21 ± 14.82 μm. Further subgroup analysis based on anti-VEGF agents showed the MAEs of post-therapeutic OCT images from patients treated with Ranibizumab and Conbercep were 25.91 ± 18.22 and 26.33 ± 13.94 μm. In addition, during the subgroup analysis of the injection phase, the MAEs of post-therapeutic OCT images from patients in the loading and PRN phases were 21.76 ± 12.35 μm and 28.56 ± 18.93 μm. Finally, the MAEs of post-therapeutic OCT images from patients with and without laser photocoagulation were 33.30 ± 21.02 and 24.80 ± 13.12 μm. Details of MAEs between synthetic OCT images and ground truth data are shown in Table 2.

**TABLE 2:** Accuracy of the synthetic post-therapeutic OCT images of RVO in the evaluating experiment.

	Baseline		1-month prediction	
CMT (μm)	Real images	Synthetic images	Real images	MAE
Validation data	389.30 ± 243.45	310.76 ± 198.23	297.34 ± 178.66	26.33 ± 15.81
Classification of RVO				
CRVO	410.34 ± 256.27	321.22 ± 208.72	309.56 ± 188.61	28.55 ± 17.32
BRVO	367.69 ± 232.76	303.24 ± 187.31	279.54 ± 176.18	24.21 ± 14.82
Anti-VEGF agent (%)				
Ranibizumab	390.15 ± 246.75	307.77 ± 201.15	302.65 ± 183.68	25.91 ± 18.22
Conbercept	386.22 ± 241.27	312.87 ± 195.29	297.34 ± 174.87	26.33 ± 13.94
Injection phase				
Loading phase	371.38 ± 228.31	297.55 ± 186.41	281.26 ± 169.08	21.76 ± 12.35
PRN phase	405.21 ± 252.64	323.82 ± 203.47	310.02 ± 186.83	28.56 ± 18.93
Combined with laser photocoagulation				
With laser photocoagulation	425.46 ± 272.28	320.52 ± 221.09	320.13 ± 190.22	33.30 ± 21.02
Without laser photocoagulation	384.21 ± 242.30	308.21 ± 191.65	280.37 ± 171.21	24.80 ± 13.12

CMT, central macular thickness; PRN, pro re nata; MAE, mean absolute error, values are presented as the means ± standard deviations.

## Discussion

Herein, we presented and evaluated the ability of a GAN-based algorithm to generate synthetic post-therapeutic OCT images to predict the structural prognosis after anti-VEGF therapy for RVO-related macular edema. Our results demonstrated that 91.18% of the synthetic OCT images were of sufficient quality for clinical interpretation, with an MAE of 26.33 ± 15.81 μm in predicting the CMT. Subgroup analysis substantiated that the anti-VEGF agents (Ranibizumab or Conbercept) had little influence on model performance. The prediction efficiency of OCT images in BRVO-related macular edema was comparable to the real ones, unlike CRVO-related macular edema. In addition, the predictions in the PRN phase were better than in the loading phase.

Macular edema is the leading cause of vision loss in RVO patients (Wong and Scott, 2010; Fogli et al., 2018; Korobelnik et al., 2021). At present, intravitreal injection of anti-VECF is established as the first-line therapy to promote structural and functional recovery of patients with RVO (Campa et al., 2016; Paciullo et al., 2021). During clinical practice, loss to follow-up is common, given the high costs of anti-VEGF therapy, the need for repeated treatment and the uncertainty of prognosis. In a cohort study carried out for 7 years, researchers found that patients with CRVO required an average of 10.70 ± 4.76 doses of anti-VEGF therapy, while patients with BRVO required 9.80 ± 5.39 doses of anti-VEGF therapy (Arrigo et al., 2021). Moreover, a study by Yang et al. showed that 41.2% of patients with RVO who discontinued follow-up for more than 6 months developed complications associated with retinal neovascularization, while all patients experienced more severe macular edema than baseline, with an average CMT of 738.7 ± 143.6 μm. Moreover, the visual acuity significantly decreased compared with the baseline. In another retrospective cohort study, patients lost to follow-up for more than 6 months lost nearly 3 Best Corrected Visual Acuity (BCVA) lines of vision, and their vision was not restored after anti-VEGF therapy (Salabati et al., 2021). In the present study, our model accurately predicted the prognosis of anti-VEGF therapy based on the pre-therapeutic OCT images. In the real world, the treatment effect of most patients is satisfactory and can relieve the psychological burden of patients, improve their follow-up compliance, reduce the possibility of treatment interruption, and thus reduce the risks of irreversible visual impairment in patients with RVO.

Prediction and evaluation of the short-term efficacy of anti-VEGF therapy are essential for clinical follow-up and management of patients with RVO. In recent years, scholars have carried out exploratory studies to bridge this knowledge gap (Ting et al., 2021). Liu et al. (2020a) successfully used pre-therapeutic OCT images to predict OCT images after anti-VEGF of patients of nAMD based on GAN, with an accuracy of 85% for predicting macular state after treatment. However, they mainly conducted a quantitative evaluation of macular types, lacking objective quantitative results of CMT. More recently, Lee et al. (2021b) developed a deep learning model to generate post-therapeutic OCT images of nAMD based on OCT, FFA, and Indocyanine Green Angiography collected at baseline, which yielded an acceptable accuracy and specificity (range: 77.0–91.9 and 94.1–95.1, respectively). However, FFA and indocyanine green angiography are widely acknowledged as invasive examinations with high equipment and technician expertise requirements. Despite their limitations, these attempts also substantiated the potential application of GAN-based algorithms in predicting the structural prognosis of ocular fundus diseases. In this study, the pix2pixHD algorithm was used to predict the effect of anti-VEGF therapy on RVO-related macular edema based on pre-therapeutic OCT images. Meanwhile, a structural assessment was carried out to evaluate post-therapeutic images qualitatively and quantitatively. Importantly, the pix2pixHD algorithm could predict the short-term response of anti-VEGF therapy with high accuracy, which could help improve the treatment compliance of RVO patients and identify patients with poor responses to treatment to optimize the treatment plan.

In this study, the anti-VEGF agents adopted were Conbercept and Lucentis. Lucentis is a 48 kDa recombinant humanized immunoglobulin G1κ isotype monoclonal antibody fragment (Fab) that binds to VEGF-A and avoids interactions with VEGFR1 and VEGFR2. Conbercept is a recombinant human IgG fusion protein composed of the second Ig domain of VEGFR1 and the third and fourth Ig domains of VEGFR2 (Fogli et al., 2018; Porta and Striglia, 2020). Overwhelming evidence suggests that Conbercept yields a better effect than Lucentis, especially in improving BCVA (Liu et al., 2020b). However, in our study, there was no significant difference in the efficacy and predicted performance between Conbercept and Lucentis. This discrepancy may be attributed to the relatively small sample size and short follow-up period. Indeed, this study focused on predicting post-therapeutic OCT images and CMT evaluation without BCVA and other functional indicators in the prediction tasks due to the insufficient sample size and the lack of feature extraction for machine learning and modality fusion. This is also a shortcoming of our model that emphasizes the need for further improvements.

Several limitations were present in this study. First, the sample size in this study was limited, which may influence the predicted performance of patients with RVO. Greater sample sizes are warranted to improve the stability of the model. Moreover, we included post-therapeutic B-scans for short-term outcomes as the follow-up visit of the enrolled patients was scheduled at 1 month ± 7 days, which limited the established model’s ability to conduct long-term predictions. However, RVO patients often require multiple types of treatment. Accordingly, the long-term outcome is what ophthalmologists and patients are most concerned about.

In conclusion, the GAN-based prediction model yielded promising results and successfully demonstrated its potential for predicting the prognosis of RVO-related macular edema. Predicting therapeutic response to anti-VEGF treatment remains challenging in clinical practice. Our pix2pixHD algorithm could accurately predict post-therapeutic OCT images 1 month after anti-VEGF therapy, providing the postoperative OCT morphology and more prognostic information, potentially improving treatment adherence and the prognosis of this patient population. Indeed, our prediction model could assist physicians in identifying patients with poor responses to treatment and optimizing the drug regimen.

## Data Availability

The original contributions presented in the study are included in the article/Supplementary Material; further inquiries can be directed to the corresponding author.
