# Supplementary material for: Predicting OCT images of short-term response to anti-VEGF treatment for retinal vein occlusion using generative adversarial network
Source: Front Bioeng Biotechnol. 2022 Oct 12;10:914964. doi: 10.3389/fbioe.2022.914964 (PMC9596772; doi:10.3389/fbioe.2022.914964)
Supplement: Supplementary file 1 [file DataSheet1.DOCX]

Figure S1. Examples of inadequate and easily distinguished synthetic images.


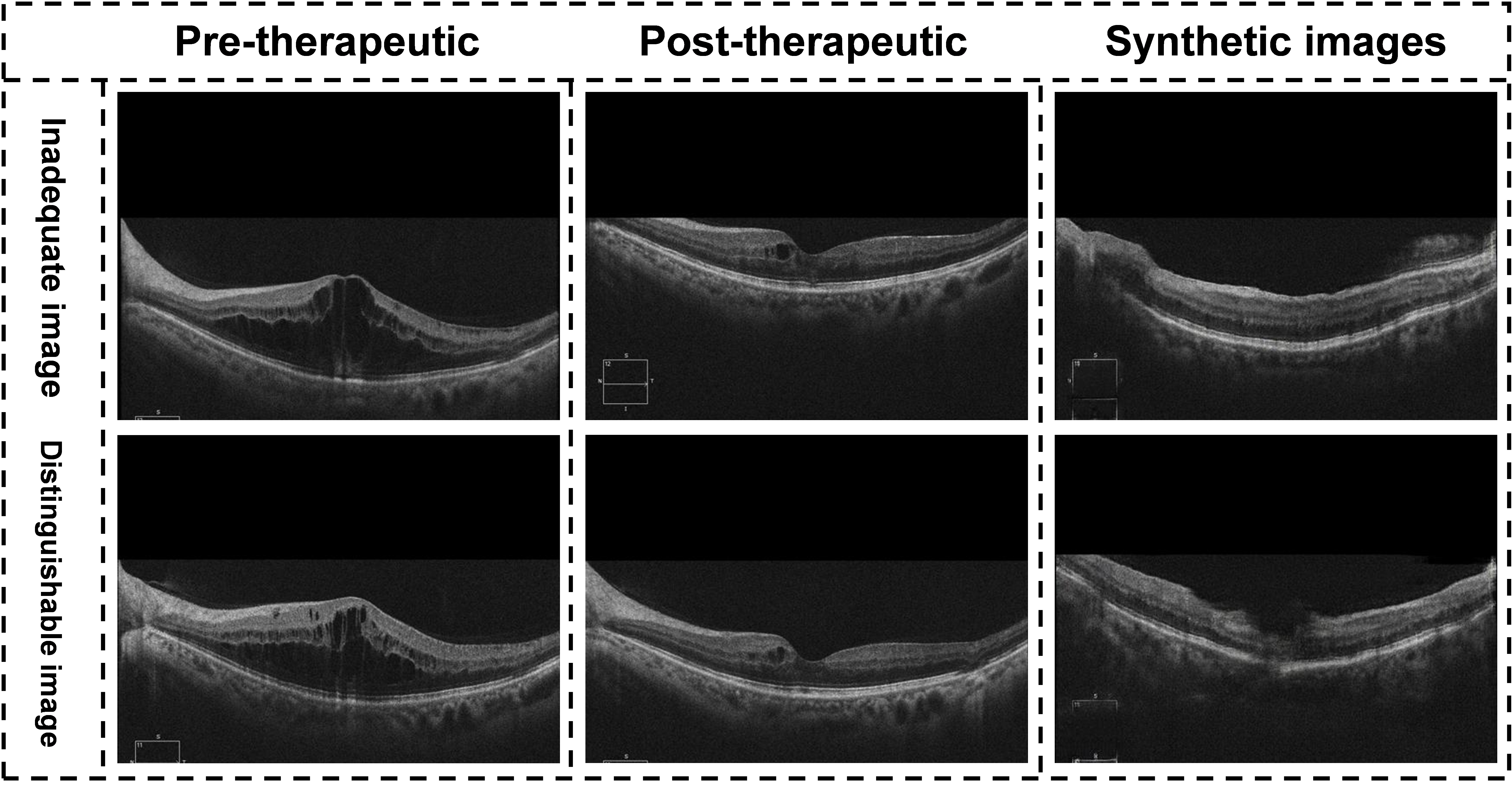


Illustration of the synthetic OCT images of different qualities. The images in the left, middle and right columns are pre-therapeutic, real post-therapeutic, and synthetic post-therapeutic images, respectively. Two retinal specialists considered the upper right image inadequate during the evaluation process since it did not reflect the actual retinal structures; the lower right image was easily distinguished by the two retinal specialists during the evaluation process.

**Table S1. S****tatistical analysis of the Synthetic Post-therapeutic OCT Images of RVO in the Evaluating Experiment**

|  | **1-mo Prediction** | | | **P values** |
| --- | --- | --- | --- | --- |
| **CMT (μm)** | Synthetic images | Real images | MAE |  |
| **Validation data** | 310.76 ± 198.23 | 297.34 ± 178.66 | 26.33 ± 15.81 | 0.674 |
| **Classification of RVO** |  |  |  |  |
| CRVO | 321.22 ± 208.72 | 309.56 ± 188.61 | 28.55 ± 17.32 | 0.257 |
| BRVO | 303.24 ± 187.31 | 279.54 ± 176.18 | 24.21 ± 14.82 | 0.109 |
| **Anti-VEGF agent (%)** |  |  |  |  |
| Ranibizumab | 307.77 ± 201.15 | 302.65 ± 183.68 | 25.91 ± 18.22 | 0.532 |
| Conbercept | 312.87 ± 195.29 | 297.34 ± 174.87 | 26.33 ± 13.94 | 0.164 |
| **Injection phase** |  |  |  |  |
| Loading phase | 297.55 ± 186.41 | 281.26 ± 169.08 | 21.76 ± 12.35 | 0.207 |
| PRN phase | 323.82 ± 203.47 | 310.02 ± 186.83 | 28.56 ± 18.93 | 0.129 |
| **Combined with laser photocoagulation** |  |  |  |  |
| With laser photocoagulation | 320.52 ± 221.09 | 320.13 ± 190.22 | 33.30 ± 21.02 | 0.856 |
| Without laser photocoagulation | 308.21 ± 191.65 | 280.37 ± 171.21 | 24.80 ± 13.12 | 0.067 |

CMT, central macular thickness; PRN, pro re nata; MAE, mean absolute error, values are presented as the means ± standard deviations. P < 0.05 indicates a statistically significant difference.
